# Vertebral artery occlusion mediated cerebellar and spinal cord infarction: A case report

**DOI:** 10.1097/MD.0000000000045428

**Published:** 2025-10-24

**Authors:** Guanqun Hu, Yanting Liu, Meiyun Zhang

**Affiliations:** a Department of Neurology, Tianjin Union Medical Center, The First Affiliated Hospital of Nankai University, Tianjin, China.

**Keywords:** cerebellar infarction, dual-territory infarction, spinal cord infarction, vertebral artery occlusion

## Abstract

**Rationale:**

This case report describes a rare instance of concurrent acute cerebellar and spinal cord infarction caused by right vertebral artery occlusion, providing dynamic angiographic visualization of the collateral circulation.

**Patient concerns:**

A 69-year-old male with hypertension and diabetes presented with thunderclap occipital headaches, left-dominant limb hypesthesia, and mild bladder dysfunction.

**Diagnoses:**

Brain magnetic resonance imaging revealed an acute right cerebellar infarction in the posterior inferior cerebellar artery (PICA) territory. Cervical magnetic resonance imaging demonstrated bilateral anterior horn hyperintensity (“owl’s eye” sign) at C2 to C4, consistent with spinal cord infarction. Digital subtraction angiography confirmed atherosclerotic occlusion at the right vertebral artery origin, with collateral circulation via the deep cervical artery. Cerebrospinal fluid analysis showed elevated protein (0.679 g/L) without pleocytosis or autoantibodies (AQP4/MOG/MBP), excluding inflammatory/demyelinating etiologies.

**Interventions:**

The patient was treated with antiplatelet therapy (aspirin) and statins (rosuvastatin).

**Outcomes:**

The patient’s symptoms significantly improved within 2 weeks (headache Visual Analog Scale score decreased from 8 to 2).

**Lessons:**

This is the first reported case combining acute cerebellar (PICA territory) and cervical spinal cord (C2–C4) infarction, with digital subtraction angiography visualization of collateral circulation via the deep cervical artery. It underscores vertebral artery occlusion as a rare yet critical cause of dual cerebellar–spinal infarction, mediated by hemodynamic compromise in both PICA and anterior spinal artery territories.

## 1. Background

Acute cerebral infarction is the most common type of stroke. Spinal cord infarction represents an uncommon neurovascular emergency, accounting for < 1% of central nervous system infarctions.^[1]^ Concurrent spinal and cerebral infarction is exceptionally rare, with only isolated case reports documented. We present a 69-year-old hypertensive and diabetic male manifesting acute right cerebellar infarction accompanied by C2 to C4 spinal cord infarction. Digital subtraction angiography (DSA) identified proximal atherosclerotic occlusion at the right vertebral artery origin. Notably, the patient’s initial presentation with thunderclap headache and myelopathic features closely mimicked inflammatory myelopathies, underscoring the diagnostic imperative for prompt vascular imaging (e.g., contrast-enhanced MRI and angiography) in dual-territory ischemic syndromes. This case highlights the diagnostic challenges and vascular mechanisms underlying dual-territory infarctions.

## 2. Case presentation

A 69-year-old man with a medical history of well-controlled hypertension and type 2 diabetes presented with a 48-hour history of recurrent thunderclap occipital headaches (paroxysmal episodes lasting < 5 seconds, frequency 10–20/day), 8/10 on the visual analog scale, accompanied by progressive left-dominant upper limb sensory impairment. Neurological examination demonstrated left-sided central facial paresis, ipsilateral hypoglossal nerve deviation, absent bilateral Achilles reflexes, and equivocal plantar response on the left (Babinski sign ±). Sensory testing revealed diminished pinprick sensation over the left C6 to T1 dermatomes. The patient denied alcohol consumption, smoking, or recreational drug use. No family history of early-onset cerebrovascular disease was documented. National Institutes of Health Stroke Scale score was 2 (1 point for facial palsy and 1 poin for sensory loss).

**Figure 1.**
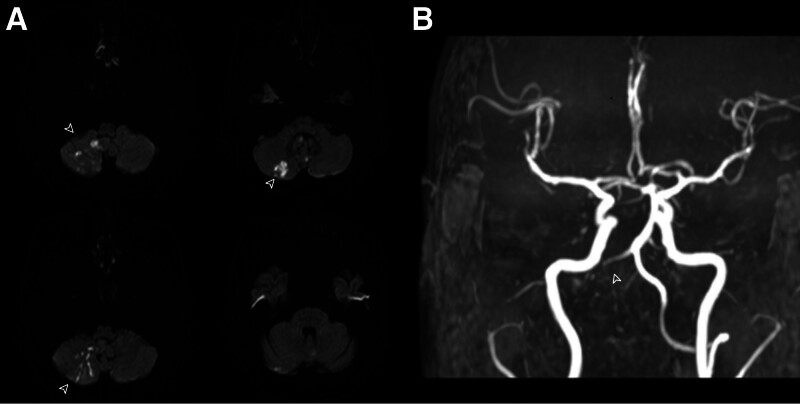
Neuroimaging of acute cerebellar infarction and vertebral artery occlusion. (A) Axial diffusion-weighted imaging (DWI; b = 1000 s/mm²): restricted diffusion in the right cerebellar hemisphere (arrow), predominantly involving the posterior inferior cerebellar artery (PICA) territory, consistent with acute infarction. (B) Magnetic resonance angiography (MRA; time-of-flight sequence): complete occlusion of the right vertebral artery (arrow).

**Figure 2.**
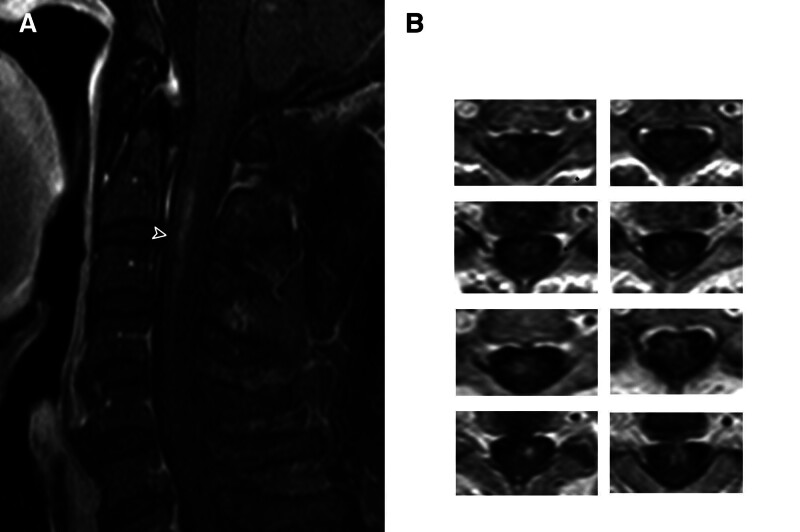
Spinal cord infarction features on contrast-enhanced MRI. (A) Sagittal T1-weighted post-contrast image: linear gadolinium enhancement along the anterior spinal artery distribution (arrows) spanning C2 to C4. (B) Axial T2-weighted image: bilateral symmetric hyperintensity in the anterior horns (“owl’s eye” sign).

Initial brain MRI demonstrated an acute right cerebellar infarction localized to the posterior inferior cerebellar artery (PICA) territory, evidenced by restricted diffusion on diffusion-weighted imaging/apparent diffusion coefficient sequences (Fig. 1A). Concurrent magnetic resonance angiography revealed complete occlusion of the right vertebral artery origin (Fig. 1B). Cervical spine MRI identified a longitudinally extensive T2 hyperintensity spanning C2 to C4. Subsequent contrast-enhanced cervical MRI clarified the diagnosis, demonstrating bilateral symmetrical hyperintensity of the anterior horns (Fig. 2A) and linear enhancement along the anterior spinal artery (ASA) distribution (“owl eyes” sign, Fig. 2B), pathognomonic of spinal cord infarction. Thoracic spine MRI (T1/T2-weighted sequences) showed no structural abnormalities, excluding compressive etiologies.

**Figure 3.**
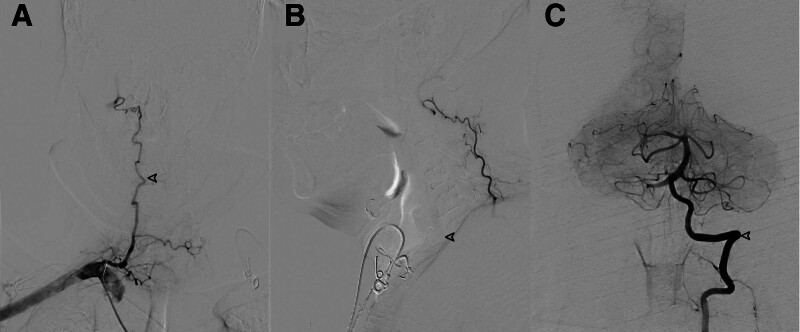
Digital subtraction angiography findings of right vertebral artery occlusion. (A) Anteroposterior view of right vertebral artery (RVA) angiography: Complete occlusion at the origin of the right vertebral artery. Collateral supply to the V3 segment via the deep cervical artery (white arrow) is observed. (B) Oblique view of RVA angiography: confirms occlusion at the vertebral artery origin, excluding vascular tortuosity or artifact (arrows). (C) Left vertebral artery (LVA) angiography: robust collateral circulation from the LVA (arrows) to the right posterior inferior cerebellar artery (PICA) territory via posterior spinal arteries.

To exclude inflammatory demyelinating etiologies (e.g., neuromyelitis optica spectrum disorder, multiple sclerosis), lumbar puncture was performed on day 7 post-admission. Cerebrospinal fluid (CSF) analysis demonstrated elevated protein without pleocytosis (protein 0.679 g/L [normal: 0.15–0.45 g/L]; glucose 5.65 mmol/L [serum glucose 9.2 mmol/L]) with normal nucleated cell count (3/μL). Immunological profiling ruled out autoantibody-mediated processes (AQP4/MOG/MBP antibodies negative in both serum and CSF) and intrathecal synthesis (absence of oligoclonal bands). DSA on day 14 confirmed atherosclerotic occlusion at the right vertebral artery origin, collateral supply to the V3 segment via the deep cervical artery was observed (Fig. 3A). Oblique view of RVA angiography confirmed occlusion at the vertebral artery origin, excluding vascular tortuosity or artifact (Fig. 3B). Left vertebral artery angiography found robust collateral circulation from the left vertebral artery to the right posterior inferior cerebellar artery (PICA) territory via posterior spinal arteries (Fig. 3C). Notably, DSA did not reveal evidence of arterial dissection or arteriovenous malformation. The patient was ultimately diagnosed with acute cerebellar infarction, acute spinal cord infarction, and vertebral artery occlusion.

Systemic evaluation revealed suboptimal glycemic control (HbA1c 8.4%) without evidence of systemic inflammation (CRP 0.8 mg/L) or autoimmune dysregulation (negative antinuclear antibodies, antineutrophil cytoplasmic antibodies). Routine hematological, biochemical (including renal and hepatic function), coagulation, and thyroid profiles were unremarkable. Complement levels remained within normal limits. Cardiac monitoring showed sinus rhythm without arrhythmias. Lower extremity venous ultrasound reported revealed muscular vein thrombosis in bilateral gastrocnemius veins.

The patient initiated guideline-directed secondary stroke prevention, including antiplatelet therapy (aspirin 100 mg/day) and statin (rosuvastatin 10 mg/day), anticoagulation with rivaroxaban 10 mg daily was initiated alongside antiplatelet therapy. Neuroprotective measures comprised glycerol fructose infusion (125 mL/day) to mitigate edema. Neuropathic pain was managed with pregabalin (75 mg twice daily), supplemented with vitamin B complex (intramuscular thiamin 50 mg/day + methylcobalamin 0.5 mg/day) to support axonal repair. Preexisting antihypertensive and antidiabetic regimens were maintained (amlodipine 5 mg once daily; metformin 500 mg 3 times daily + acarbose 50 mg 3 times daily).

By day 16 of admission, the patient achieved clinically significant improvement: headache severity decreased from 8/10 to 2/10 on the visual analog scale, with resolution of sensory deficits. The patient was discharged with structured rehabilitation and close glycemic monitoring.

## 3. Discussion

Spinal cord infarction (SCI) represents < 1% of all central nervous system infarctions, with an annual incidence of 3 to 12 cases per million, far rarer than cerebral infarction.^[1]^ The thoracic segment (T4–T8) is most commonly affected (50%), followed by the cervical region (C2–C4, 30%), the latter often linked to vertebral artery pathology. Atherosclerosis underlies 80% of SCI cases, while hypoperfusion, hypercoagulable states, and vascular malformations account for the remainder.^[2]^ Clinically, SCI manifests as acute-onset motor deficits, dissociative sensory loss (spinothalamic tract involvement with preserved proprioception), and sphincter dysfunction.^[3]^ Unusual presentations, such as radiculopathy-like symptoms, further complicate diagnosis. A rare case^[4]^ showed that motor symptoms mimicking a radiculopathy could be present during the course of spinal cord infarction. Concurrent cerebellar and spinal cord infarction is exceedingly rare, with limited reported cases. A case in 2023 reported a combined posterior spinal cord and ipsilateral cerebellar infarction,^[5]^ another case in 2022 reported a long-segment spinal cord infarction complicated with multiple cerebral infarctions.^[6]^ However, neither case included lumbar puncture to exclude inflammatory mimics nor DSA examination.

In this case, occlusion at the right vertebral artery origin disrupted perfusion to both the PICA and ASA. The ASA, arising from vertebral artery branches, supplies the anterior two-thirds of the spinal cord. Occlusion at its origin likely caused ischemia in the ASA-dependent cervical cord (C2–C4), explaining the bilateral anterior horn “owl’s eye” sign on MRI: a hallmark of ASA territory infarction.^[7,8]^ This sign reflects ischemic necrosis of metabolically active anterior horn neurons in SCI. Crucially, the absence of CSF pleocytosis, autoantibodies (AQP4/MOG/MBP), and demyelinating markers differentiated this case from myelitis, while DSA confirmed the vascular etiology. The absence of arterial dissection or arteriovenous malformation on DSA further supports atherosclerosis as the primary etiology. These findings emphasize the indispensability of multimodal imaging (MRI + angiography) and CSF analysis in distinguishing vascular from inflammatory lesions.

This case exemplifies 2 diagnostic pitfalls. First, the patient’s occipital headache and sensory symptoms initially mimicked intracranial hypertension or myelitis. Similar ambiguity was reported in a case of C2 SCI masquerading as occipital neuralgia,^[9]^ highlighting the need to exclude secondary causes of cephalic pain, particularly in high-risk populations. Second, dual cerebellar and spinal infarcts may obscure localization, as symptoms span posterior circulation and spinal pathways. For instance, limb numbness could be misattributed to cerebellar dysfunction rather than concurrent spinothalamic tract injury. Such overlap underscores the importance of systematic neurological examination and targeted imaging, especially when symptoms defy a single anatomical locus.

The patient’s hypertension and diabetes likely accelerated atherosclerotic plaque formation at the vertebral artery origin, a vulnerable site for flow-limiting stenosis. While antiplatelet, anticoagulation therapy and statins stabilized his condition, endovascular intervention remains an option for refractory cases or recurrent symptoms. Early differentiation from vertebral dissection and hypercoagulable states is critical, as management diverges radically. Notably, thunderclap headache and abrupt symptom onset favored vascular etiology over inflammatory myelitis, prompting timely antiplatelet initiation to mitigate thrombus propagation. This aligns with emerging evidence supporting antiplatelet therapy in acute vertebral artery occlusion to prevent downstream embolism.

The co-occurrence of cerebellar and spinal infarction exposes the anatomical vulnerability of vertebrobasilar-dependent territories. The right vertebral artery’s dual role in supplying both PICA and ASA creates a “watershed” scenario when collateral circulation is inadequate. This case challenges the traditional view of spinal and cerebral infarctions as distinct entities, proposing a continuum of vertebrobasilar insufficiency syndromes. Future studies should explore genetic or hemodynamic predispositions to multi-territory infarcts and validate imaging protocols (e.g., combined brain–spinal MRI + DSA) for early detection. Additionally, the prognostic significance of “owl’s eye” signs in SCI warrants investigation, as anterior horn damage may correlate with long-term motor recovery.

## 4. Conclusion

This case illuminates the intricate interplay between vascular anatomy, imaging findings, and clinical phenotypes in dual cerebellar–spinal infarction. It reinforces the necessity of holistic vascular evaluation in patients with overlapping posterior circulation and spinal symptoms, particularly those with atherosclerotic risk factors. Clinicians should prioritize vascular imaging when cerebellar stroke coexists with spinal symptoms, even if inflammatory mimics are initially suspected. While MRI remains indispensable for detecting acute spinal cord lesions, DSA should be regarded as the gold standard for suspected vertebral artery pathology, particularly when MRI reveals multi-territorial ischemia. DSA remains indispensable not only for confirming atherosclerotic occlusion but also for excluding alternative pathologies such as dissection or vascular malformations.

## Author contributions

**Conceptualization:** Meiyun Zhang.

**Data curation:** Guanqun Hu, Meiyun Zhang.

**Funding acquisition:** Guanqun Hu, Yanting Liu, Meiyun Zhang.

**Methodology:** Yanting Liu.

**Supervision:** Meiyun Zhang.

**Writing – original draft:** Guanqun Hu, Meiyun Zhang.

**Writing – review & editing:** Guanqun Hu, Meiyun Zhang.
